# Diagnosis and treatment of cervical cancer during pregnancy

**DOI:** 10.1590/S1516-31802009000600008

**Published:** 2010-05-21

**Authors:** Carla Vitola Gonçalves, Geraldo Duarte, Juvenal Soares Dias da Costa, Alessandra Cristina Marcolin, Mônia Steigleder Bianchi, Daison Dias, Luis Cláudio de Velleca e Lima

**Affiliations:** I MD. Adjunct professor, Mother-Child Department, Universidade Federal do Rio Grande (FURG), Rio Grande, Rio Grande do Sul, Brazil; II MD, PhD. Titular professor, Department of Gynecology and Obstetrics, Faculdade de Medicina de Ribeirão Preto (FMRP), Universidade de São Paulo (USP), Ribeirão Preto, São Paulo, Brazil.; III MD. Adjunct professor, Department of Social Medicine, Universidade Federal de Pelotas (UFPel), Pelotas, Rio Grande do Sul, Brazil.; IV MD. Gynecologist and Obstetrician, Hospital das Clínicas (HC), Faculdade de Medicina de Ribeirão Preto (FMRP), Universidade de São Paulo (USP), Ribeirão Preto, São Paulo, Brazil.; V Undergraduate medical student, Universidade Federal do Rio Grande (FURG), Rio Grande, Rio Grande do Sul, Brazil.; VI Undergraduate medical student, Universidade Federal de Pelotas (UFPel), Pelotas, Rio Grande do Sul, Brazil.

**Keywords:** Uterine cervical neoplasms, Uterine cervical dysplasia, Diagnosis, Treatment effectiveness, Treatment protocols, Pregnancy, Neoplasia do colo do útero, Displasia do colo do útero, Diagnóstico, Resultado de tratamento, Protocolos clínicos, Gravidez

## Abstract

**CONTEXT AND OBJECTIVE:**

One third of all cervical carcinomas occur during the reproductive period. Cervical carcinoma is the second greatest cause of death due to cancer during this phase. The estimated frequency of cervical cancer during pregnancy is one case for every 1,000 to 5,000 pregnancies. The aim here was to provide information about the difficulties in diagnosing and managing cervical neoplasia during pregnancy.

**MATERIALS:**

A systematic review of the literature was undertaken through the PubMed, Cochrane, Excerpta Medica (Embase), Literatura Latino Americana e do Caribe em Ciências da Saúde (Lilacs) and Scientific Electronic Library Online (SciELO) databases, using the following words: pregnancy, cervical cancer, diagnosis and management.

**RESULTS:**

There was a consensus in the literature regarding diagnosis of cervical carcinoma and management of preneoplastic lesions during pregnancy. However, for management of invasive carcinoma, there was great divergence regarding the gestational age taken as the limit for observation rather than immediate treatment.

**CONCLUSION:**

All patients with cytological abnormalities should undergo colposcopy, which will indicate and guide biopsy. Conization is reserved for patients with suspected invasion. High-grade lesions should be monitored during pregnancy and reevaluated after delivery. In cases of invasive carcinoma detected up to the 12^th^ week of pregnancy, patient treatment is prioritized. Regarding diagnoses made during the second trimester, fetal pulmonary maturity can be awaited, and the use of chemotherapy to stabilize the disease until the time of delivery appears to be viable.

## RESUMO

**CONTEXTO E OBJETIVO:**

Um terço dos carcinomas de colo ocorrem no período reprodutivo, sendo que esta é a segunda causa de morte por câncer nessa fase. A freqüência estimada do carcinoma de colo uterino na gravidez é de um caso para cada 1.000 a 5.000 gestações. O objetivo foi informar sobre as dificuldades frente ao diagnóstico e manejo da neoplasia cervical durante a gravidez.

**MATERIAIS E MÉTODOS:**

Revisão sistemática da literatura foi realizada no PubMed, Cochrane, Excerpta Medica (Embase), Literatura Latino Americana e do Caribe em Ciências da Saúde (Lilacs) and Scientific Electronic Library Online (SciELO), usando as seguintes palavras: *gestação, câncer cervical, diagnóstico e manejo*.

**RESULTADOS:**

A literatura apresenta consenso quanto ao diagnóstico do carcinoma cervical e a conduta das lesões pré-neoplásicas durante a gestação. No manejo do carcinoma invasor há grande divergência quanto à idade gestacional considerada como limite para a adoção da observação em vez do tratamento imediato.

**CONCLUSÃO:**

Toda paciente com citologia alterada deve realizar colposcopia, a qual indicará e a biópsia. A conização é reservada para pacientes com suspeita de invasão. As lesões de alto grau devem ser acompanhadas durante a gestação e reavaliadas após o parto. Em casos de carcinoma invasor em gestantes com até 12 semanas o tratamento da paciente é priorizado. Nos diagnósticos ocorridos no segundo trimestre, pode-se aguardar a maturidade pulmonar fetal e o uso da quimioterapia para estabilizar a doença até o momento do parto parece ser viável.

## INTRODUCTION

One third of all cervical carcinomas occur during the reproductive period.^1^^,^^2^ This type of neoplasia is the second greatest cause of death due to cancer, only preceded by breast cancer of the breast. It is followed in frequency by lymphomas, melanoma and thyroid carcinoma.^3^^,^^4^

About 3% of cases of cervical cancer are diagnosed during pregnancy,^5^ and these cases correspond to half of the cases of neoplasia diagnosed during the gestational period. Cervical cancer is the most frequently found malignancy worldwide. The estimated frequency is one case per 1,000 to 5,000 pregnancies.^5^^,^^6^

Current evidence indicates that the chance that pregnant women will be diagnosed with cervical cancer while it is in its initial stages is three times greater than the chance among controls. This is due to inspections and cervical cytological tests conducted among women in countries where these examinations are part of routine prenatal care. Several studies have shown that 76% of the lesions diagnosed during pregnancy are in stage IB^4^^,^^5^^,^^6^^,^^7^^,^^8^^,^^9^^,^^10^^,^^11^ (Table 1).

Although diagnoses of cervical cancer during pregnancy are usually made while the disease stage is operable, this time does not always coincide with fetal maturity, thus causing anguish for both the patient and the medical team. The aim of the present paper was to provide information about the difficulties in diagnosing and managing cervical cancer during pregnancy.

**Table 1.** Comparison of staging at the time of diagnosing cervical cancer in pregnant and non-pregnant women^9^^,^^56^

Staging	Pregnant	Non-pregnant
Stage I	70-80%	42%
Stage II	11-20%	35%
Stage III	3-8%	21%
Stage IV	0-3%	2%

## MATERIALS

The present review discusses the current guidelines for diagnosis and management of cervical cancer associated with pregnancy. A systematic review of the literature was undertaken by searching in the PubMed, Cochrane, Excerpta Medica (Embase), Literatura Latino Americana e do Caribe em Ciências da Saúde (Lilacs) and Scientific Electronic Library Online (SciELO) databases. The study was developed using the following key words: pregnancy, cervical cancer, diagnosis and management. The search strategies and the results are shown in Table 2. Articles published since 1996 were selected according to the following criteria: literature reviews, prospective studies and case reports presenting approaches for diagnosing and managing cancer of the uterine cervix during pregnancy, particularly using innovative treatments, protocols and consensuses from institutions specializing in this disease. Articles reporting on diagnosis and management of cervical cancer among non-pregnant women and articles that evaluated the prognosis for a future pregnancy when comparing the various types of treatment for this disease were excluded from this review.

**Table 2.** Systematic review of the literature, searching for papers published over the last 13 years (1996-2009)

Database	Strategy of search	Results
PubMed	Pregnancy [MeSH] AND cervical cancer [MeSH] AND diagnosis [MeSH] AND management [MeSH]	324 articles 91 review articles 86 management articles 64 screening articles 29 cross-sectional prevalence studies 25 guidelines 15 clinical trials 14 case histories
Cochrane	Pregnancy [MeSH] AND cervical cancer [MeSH] AND diagnosis [MeSH] AND management [MeSH]	41 articles 37 systematic reviews 2 clinical trials 2 economic evaluations
Embase (Excerpta Medica Database)	Pregnancy [MeSH] AND cervical cancer [MeSH ] AND diagnosis [MeSH] AND management [MeSH]	20 articles 10 guidelines 5 systematic reviews 3 cross-sectional prevalence studies 2 clinical trials
Lilacs (Literatura Latino-Americana e do Caribe em Ciências da Saúde)	Gestação [DeCS] AND câncer cervical [DeCS] AND diagnóstico [DeCS] AND tratamento	2 articles 1 case history 1 systematic reviews
SciELO (Scientific Electronic Library Online)	Gestação [DeCS] AND câncer cervical [DeCS] AND diagnóstico [DeCS] AND tratamento	11 articles 9 case histories 2 systematic reviews

MeSH = Medical Subjects Headings; DeCS = Descritores em Ciências da Saúde.

## DIAGNOSIS

The diagnosis of cervical carcinoma in pregnant women is based on clinical findings, inspection of the uterine cervix, cytological tests, colposcopy, directed biopsy and imaging examinations.^9^

### Clinical findings

In most cases, patients with stage I cervical carcinoma are asymptomatic at the time of diagnosis. When symptomatic, they report yellowish, fetid or bloody vaginal content, postcoital bleeding and vague pain in the hypogastrium. At more advanced stages, patients may report lumbar pain, hematuria, changes in micturition and changes in intestinal habitus.^5^^,^^9^ The most prevalent symptom during pregnancy is vaginal bleeding, which is present in 50% of the cases.^10^

### Inspection of the uterine cervix

The uterine cervix undergoes general modification during pregnancy due to the physiological and hormonal changes that occur during this period. The cervix may double or almost triple in size by the end of pregnancy and the transformation zone becomes exuberant due to eversion of the squamocolumnar junction, thus enabling better viewing. These changes may be confused with neoplastic lesions.^5^^,^^12^

### Cytology

There is no difference in the incidence of neoplastic cytopathological changes between pregnant and non-pregnant women, and these changes may reach 8%. It has been estimated that 1.2% of the patients with an abnormal Papanicolaou test have cervical carcinoma.^13^^,^^14^

Studies have shown that the efficacy of cervical cytological tests during the gestational period is the same as during non-gestational periods, and such procedures are recommended without restrictions as a screening method for lesions of the uterine cervix during pregnancy.^15^ However, it is extremely important that the physician performing the cytological tests should inform the cytologist that this material was collected from a pregnant woman, because of the physiological changes in cervical cytology that occur during pregnancy (hyperplasia of the glandular epithelium, presence of decidual cells and Arias-Stella reaction). If not informed, less experienced cytopathologists may interpret these changes as indicating intraepithelial lesions.^9^^,^^14^^,^^16^^,^^17^

Another important event is eversion of the endocervical epithelium, which causes the transformation zone to be more exposed to physical trauma (sexual intercourse), infections or vaginal pH trauma. All of these elements generate reparative reactions (immature metaplasia) that may be confused with cell atypia of the glandular epithelium and may increase the rate of diagnosing preneoplastic lesions of the cervix. In experienced laboratories, the incidence of these changes is similar for pregnant and non-pregnant women, about 0.6%.^14^^,^^16^

### Colposcopy

Any pregnant woman with abnormal cytological test results should be referred for colposcopy. This, in addition to identifying suspected neoplastic lesions, indicates the most appropriate site for a biopsy. This procedure makes it possible to rule out or confirm the presence of stromal microinvasion or invasion, thereby defining the type of treatment and the time when it should be applied, i.e. before or after delivery.^9^

During pregnancy, the fundamental characteristics of the colposcopic examination do not differ from those among non-pregnant women. Examinations with unsatisfactory results are uncommon, since the squamocolumnar junction is visible in 90 to 100% of all pregnant women.^18^ The professional responsible for the colposcopic examination should be experienced in examining pregnant women, because the increased cervical volume, stromal edema and hyperplasia of the glandular epithelium give rise to greater mucus production, and the decidual reaction may impair accomplishment of the examination.^13^^,^^19^ A vaginal speculum examination or evaluation of the cervix by quadrants may be necessary. It is also important to point out that the increased vascularization of the uterine cervix and the prominent reaction of the metaplastic epithelium to acetic acid may lead to a suspicion of a more severe lesion that is confirmed by histopathological examination.^9^ Thus, inspection of the cervix and colposcopy alone are insufficient to distinguish premalignant lesions from invasive lesions, whereas a biopsy directed by colposcopy is the most sensitive method.^9^^,^^17^^,^^19^^,^^20^

### Biopsy

Among pregnant women, the sensitivity and specificity of directed biopsies, in relation to the final diagnosis of the lesion, are 83.7% and 95.9%, respectively. When cervical biopsies are obtained during pregnancy, the risk of bleeding requiring greater accuracy of control is only 1 to 3%. Occurrences of other complications such as preterm labor and chorionic amniorrhexis are rare.^17^^,^^19^

Conization is reserved for pregnant women with suspected invasion, since this diagnosis changes the management to be followed during pregnancy in cases in which cytological tests reveal a high-grade lesion and colposcopy is unsatisfactory. However, in most cases, conization is postponed to the postpartum period.^7^^,^^20^^,^^21^^,^^22^^,^^23^ Since most of the lesions in pregnant women are located in the ectocervix, undertaking a short cone is more appropriate since this procedure is not indicated as a treatment but, rather, as a diagnostic method, because of the high rates of maternal and fetal complications. In addition, a high incidence of residual disease is observed in about half the cases.^12^

The most frequent complications from conization during pregnancy are hemorrhage (5% in the first and second trimesters and 10% in the third), abortion (25%), preterm labor (12%) and infection (2%). The risks of abortion and bleeding are considerably reduced when conization is performed during the second trimester, preferentially between the 14^th^ and 20^th^ weeks.^12^^,^^13^^,^^21^

A study on 26 pregnant women for whom there was discordance regarding the presence of an invasive lesion between the biopsy and colposcopy showed that the use of CO_2_ laser conization after the 18^th^ week of pregnancy was not associated with maternal or neonatal complications. All deliveries occurred at term and most of them were via the vaginal route. In addition, it was reported that 92.3% of the patients did not present recurrence of the lesion, after 18 months of follow-up.^24^ More studies on the use of CO_2_ laser during pregnancy are needed, although this seems to be a less risky technique for both the patient and the fetus. However, the duration of the surgery is known to be longer when the procedure is performed with a CO_2_ laser, compared with a high-frequency device. Although the results have been satisfactory, vaporization with CO_2_ cannot be taken to be the management of choice in these cases, but only as an additional therapeutic option.

Endocervical curettage is contraindicated during pregnancy because of the risk of premature rupture of the membranes and preterm delivery.^9^^,^^10^

### Imaging examinations

In general, the doses of radiation involved in imaging diagnostic procedures are much lower than the threshold for adverse effects and are not associated with fetal death, malformations or mental retardation,^25^ which may occur when the absorbed dose is higher than 10 or 15 cGy.^4^ The dose to the fetus resulting from standard examinations is lower than 0.01 Gy or, if expressed as cGy, it is equal to 1 cGy.^26^ For tomography of the abdomen or pelvis, the dose is 920 mGy or 650 mGy, respectively.^27^

However, since much relevant information can be obtained using other imaging methods, efforts are made to avoid radiography and tomography during pregnancy. Although abdominal and pelvic ultrasonography and magnetic resonance are not part of the International Federation of Gynecology and Obstetrics (FIGO) staging, these are considered to be the methods of choice for staging of pregnant women. Ultrasound can be used to evaluate kidneys and ureters, while magnetic resonance is used to assess tumor size and expansion to adjacent organs (parametrium, vagina, bladder and rectum), and for detection of lymph node metastases.^4^^,^^9^^,^^28^

Since no definitive conclusions regarding deleterious effects in fetuses or embryos exposed to magnetic resonance have been reached, the use of this technique is not recommended during the first trimester of pregnancy.^9^^,^^29^

## TREATMENT

Precursor lesions of cervical cancer should be monitored during pregnancy using cytology and colposcopy performed at three to six-month intervals. The patients should be reevaluated between six and eight weeks after delivery using the same methods, i.e. cytology and colposcopy, with a biopsy in cases requiring immediate treatment. A new biopsy during gestation is indicated only when there is cytological and colposcopic suspicion of progression to invasive disease.^7^^,^^10^^,^^11^^,^^12^^,^^13^^,^^17^^,^^20^^,^^21^^,^^22^^,^^30^^,^^31^^,^^32^

Studies involving monitoring and observation of patients with intraepithelial lesions have shown that these procedures form a safe option, such that only a very low percentage of cases progress to more severe lesions. Low-grade lesions regress in 48 to 62% of cases, while in 29 to 38% of cases they remain unchanged.^13^^,^^22^^,^^33^ Progression to more severe lesions is infrequent: no cases were observed in one series and a 6% rate was observed in another.^13^^,^^33^

For high-grade lesions, the percentages are different because the regression rate is lower, ranging from 27.4 to 34.2%, while progression of the lesion occurs in 2.7 to 9.7% of the cases. Moreover, 40.3% to 63.1% of the patients reevaluated after delivery show persistence of the lesion.^22^^,^^30^ Thus, expectant management followed by postpartum evaluation is the procedure most indicated for *in situ* carcinoma diagnosed during pregnancy, even though there is a small risk of worsening of the lesions.

## INVASIVE CERVICAL CARCINOMA

Pregnancy and cervical carcinoma occurring concomitantly causes therapeutic and ethical dilemmas. The management for this situation will depend on the gestational age at the time of diagnosis, disease staging, size of the lesion and the patient’s wish to maintain pregnancy and fertility, although the possibility of the use of *in vitro* fertilization exists.^9^

The use of steroids during pregnancy and artificial surfactants for neonates has drastically improved the prognosis for preterm newborn infants. With these procedures, neonatal intensive care units have started to have significant success with most neonates born after 24 weeks of gestation. This, taken together with the increasing number of studies suggesting that postponement of treatment of cervical cancer in pregnant women is a safe approach, has led to the use of greater caution regarding the time of delivery.^10^

Among the maternal and neonatal results for pregnant women with cervical carcinoma, one study observed that their chance of requiring delivery by cesarean section was twice as high and their chance of requiring blood transfusion was nine times as high as among those without cancer. Nevertheless, their risk of postpartum death was no greater. The neonates had twice the chance of being premature and seven times the chance of neonatal death. In addition, both the patients and newborns required prolonged hospital stay, thus involving higher costs for the healthcare system.^34^

During pregnancy, the histopathological type of cervical carcinoma is most frequently the squamous cell type (responsible for 80% of the lesions), followed by adenocarcinoma. These proportions are similar to those observed among non-pregnant women. According to FIGO, the stage distribution at the time of diagnosis shows that 70 to 80% of pregnant women with cervical cancer are in stage I, 11 to 20% in stage II, 3 to 8% in stage III and 0 to 3% in stage IV.^1^^,^^35^^,^^36^

### Microinvasive carcinoma (stage IA1)

There is no consensus regarding the treatment for microinvasive cervical carcinoma during pregnancy. Some authors have recommended conization only if the initial biopsy shows microinvasion, in order to rule out the diagnosis of clearly invasive carcinoma. When a microinvasive tumor is diagnosed, the approach consists of observation, with colposcopy at two-month intervals during the prenatal period and reevaluation six weeks after delivery using cytology and colposcopy, with a new biopsy in cases requiring immediate treatment.^37^ If the diagnosis is made after the 24^th^ week, the best approach would be to wait for fetal pulmonary maturity because of the risks of bleeding and preterm labor.^9^^,^^22^ If the surgical margins after conization are tumor-free, the patient is considered to have been cured, but should still undergo colposcopy at two-month intervals during the prenatal period and reevaluation using cytology, colposcopy and possibly biopsy six to eight weeks after delivery.^5^^,^^9^

However, other investigators have suggested that the best approach for microinvasive carcinoma diagnosed during pregnancy would be observation (without conization), with colposcopy at one or two-month intervals during prenatal care and reevaluation six weeks after delivery using cytology and colposcopy, with biopsy in cases requiring immediate treatment.^2^^,^^10^

### Invasive carcinoma (stages IA2, IB and IIA)

There is no evidence that pregnancy accelerates the natural history of cervical cancer. In addition, disease-specific survival is independent of the trimester of pregnancy during which the diagnosis is made. There is evidence supporting immediate patient treatment if the diagnosis is made before the 16^th^ week of pregnancy. If the diagnosis is made after this period, the patients with early stage tumors (IA, IB and IIA) can wait for fetal pulmonary maturity before starting treatment.^38^^,^^39^

There is wide divergence regarding the gestational age that should be considered as the limit for taking an observational approach instead of administering immediate treatment, ranging from the end of the first trimester to the 20^th^ week.^6^^,^^39^ However, the evidence shows that postponement of treatment may extend for long periods of time. All studies reporting the result from postponement of treatment after the 16^th^ week of pregnancy, in order to obtain fetal maturity, have shown that the maternal prognosis was not affected.^1^^,^^2^^,^^9^ These studies included approximately 80 cases in which treatment was postponed for periods ranging from one to 40 weeks. The recurrence rate was 5%, i.e. similar to that obtained among non-pregnant women.^1^^,^^2^^,^^3^^,^^4^^,^^6^^,^^38^^,^^39^

However, it is of fundamental importance to explain to the mother what the potential risks of treatment are and what options are available, which always include the immediate termination of pregnancy.

### Pregnancy of less than 20 weeks

In these cases, the choice is immediate and definitive treatment by means of radical hysterectomy with the fetus *in situ*^40^ and lymphadenectomy, or external radiotherapy with the fetus *in situ*, which in most cases will lead to spontaneous abortion. If this does not occur, it will be necessary to empty the uterine cavity before performing complementary brachytherapy.^2^^,^^4^^,^^9^^,^^10^^,^^35^ In young patients, radical surgery is the treatment of choice, which makes it possible to maintain functioning ovaries and to evaluate the real extent of the disease towards the parametria and ganglia. Although surgery is associated with morbidity such as bladder dysfunction and infections, these can be quickly resolved. Radiotherapy is associated with long-term complications that may even arise as much as five years after treatment.^39^ Among the most frequent complications are vaginal fibrosis, cystitis and enteritis.

Some investigators have suggested that postponement of treatment until the occurrence of fetal pulmonary maturity can be considered to be a second choice for patients with more than 12 weeks of gestation at the time of diagnosis.^4^^,^^9^^,^^10^^,^^11^^,^^35^^,^^36^

### Pregnancy of more than 20 weeks

Most studies suggest emptying of the uterine cavity by cesarean section followed by radical hysterectomy with lymphadenectomy. Neoadjuvant chemotherapy based on cisplatin can be applied in cases of locally advanced disease, followed by surgery.^2^^,^^4^^,^^5^^,^^10^^,^^41^

When a diagnosis is made after the 25^th^ week of gestation, the approach of choice is to wait for fetal pulmonary maturity before performing a cesarean section, followed by radical hysterectomy with lymphadenectomy, or chemotherapy plus radiotherapy.^2^^,^^4^^,^^10^ In patients with term gestation, the treatment is immediate.^5^^,^^9^^,^^10^^,^^35^

Despite the larger uterine volume, the results relating to factors such as radical hysterectomy, dissection of the parametria, ureteral mobilization and blood loss do not differ significantly from those observed among non-pregnant women.^5^

Some studies have suggested that chemotherapy should be used during the second trimester of pregnancy in order to make it possible to wait for fetal pulmonary maturity, with definitive treatment applied after delivery.^25^^,^^42^^,^^43^^,^^44^^,^^45^

Caluwaerts et al. reported the case of a woman in the 17^th^ week of pregnancy with a diagnosis of stage IB1 cervical carcinoma who was treated with cisplatin (six cycles of 75 mg/m^2^ for 10 days). She then underwent cesarean section followed by radical hysterectomy and lymphadenectomy at 32 weeks of gestation, without adjuvant therapy. After a follow-up of 10 months, the patient did not present recurrence of the disease.^43^

In 2007, Bader et al. reported the use of four cycles of cisplatin (50 mg/m^2^) and vincristine (1 mg/m^2^) for 21 days, for a woman in the 19^th^ week of pregnancy presenting stage IIA invasive cervical carcinoma. The patient underwent cesarean section at 33 weeks of pregnancy, with radical hysterectomy and lymphadenectomy. Since the patient had lymph nodes with metastases, she underwent three cycles of cisplatin (50 mg/m^2^), vincristine (1 mg/m^2^) and bleomycin (25 mg/m^2^) for 10 days. The patient was followed up for 80 months and did not present recurrence of the disease.^44^

Also in 2007, a report was published on the use of cisplatin starting during the 24^th^ week of gestation, among patients with stage IB2 cervical carcinoma. Cesarean section was performed during the 33^rd^ week, plus radical hysterectomy with pelvic and para-aortic lymphadenectomy and ovarian transposition, followed by pelvic radiotherapy and a new cisplatin cycle four weeks after surgery.^28^

The use of chemotherapy for treatment of cervical carcinoma during pregnancy seems to be feasible. However, the drugs involved are potent teratogens and should be avoided during the first trimester. The main fetal effects from administration of therapeutic agents during the second and third trimesters relate to low birth weight (observed in 40% of the exposed neonates), intrauterine growth restriction, prematurity and intrauterine fetal death. Furthermore, chemotherapy should not be performed beyond 35 weeks of gestation, since delivery may occur during the period of greatest fetal immunosuppression.^4^^,^^7^^,^^9^^,^^24^^,^^43^^,^^44^^,^^45^

### Invasive carcinoma (stages IIB, III and IV)

Fortunately, these stages of cervical carcinoma rarely occur during pregnancy. The literature suggests that immediate treatment should be implemented, consisting of chemotherapy based on the use of cisplatin followed by radiotherapy. It has been shown that this combination leads to a 12% increase in five-year survival, compared with using radiotherapy alone.^9^^,^^37^ Regarding diagnoses made during the second trimester of gestation, some studies have raised the hypothesis of waiting for fetal pulmonary maturity, followed by cesarean section, chemotherapy and radiotherapy after delivery. This is the approach of choice in cases diagnosed when the fetus is at term.^2^^,^^5^^,^^10^

### Preservation of fertility

Preservation of fertility in patients with cancer has become an important part of healthcare because of the growing survival rates after treatment and the postponement of maternity, especially among women from Western countries. The techniques offered for preservation of fertility are oocyte cryopreservation, ovary cryopreservation and ovary transposition. Oocyte preservation is an established technique with good rates of success, but is limited to women who do not require immediate cancer treatment, since it requires a delay in treatment of up to six weeks. For patients who cannot wait for treatment, a more recent procedure involves cryopreservation of one or both ovaries, which can be transplanted at a later time. Ovary transposition to areas far from the site of radiotherapy allows subsequent retrieval of oocytes for *in vitro* fertilization.^46^^,^^47^^,^^48^^,^^49^^,^^50^^,^^51^

## DELIVERY ROUTE

Women with a diagnosis of precursor lesions for cervical cancer or with cervical carcinoma during the gestational period should be monitored by a multidisciplinary team consisting of an obstetrician, an oncogynecologist, a psychologist and nurses experienced with this disease. In addition, such patients should be treated at reference centers for both cervical cancer and high-risk pregnancies, since delivery should be performed in accordance with the indications of their diagnosis, thus often contributing towards the treatment. The importance of concomitant presence of a neonatology center with an intensive care unit for these neonates should also be emphasized. Since they are often premature, with intrauterine growth restriction and consequent low birth weight, these infants born to mothers with a diagnosis of cervical carcinoma during pregnancy are at high risk of neonatal death.^10^^,^^33^^,^^52^^,^^53^^,^^54^^,^^55^

The choice of delivery route is based on the type and grade of the lesion. In the presence of precursor lesions for cervical carcinoma, whether of low or high grade, vaginal delivery is not contraindicated. On the contrary, some authors have correlated spontaneous regression of the lesions during the puerperium to the epithelial desquamation that occurs during delivery, associated with an improved immunological response.^10^^,^^13^^,^^52^^,^^53^

For cases with microinvasive carcinomas, the vaginal route can be chosen when conization with disease-free surgical margins is performed.^5^ However, other studies have indicated that cesarean section is the best approach.^9^^,^^10^

For patients with invasive carcinomas, the delivery route of choice is cesarean section. Vaginal delivery may result in the risk of lymphovascular dissemination of the diseases, excessive bleeding, obstruction of the birth canal, laceration of the cervix and implantation of malignant cells at the site of episiotomy. In addition, cesarean section enables complementary surgical treatment of the neoplasia when indicated. During cesarean section, corporal incision in the uterus is preferred. The placenta should be extracted and hysterorrhaphy should be performed, followed by hysterectomy.^4^^,^^9^^,^^10^^,^^53^

Few cases of metastasis in the episiotomy after vaginal delivery have been reported. The treatment recommended in the literature is surgical removal of the lesion followed by radiotherapy.^9^^,^^10^^,^^52^

## PROGNOSIS

Some studies on precursor lesions for cervical carcinoma diagnosed during pregnancy have reported that 10 to 70% regress and disappear after delivery, while 25 to 47% persist and 3 to 30% progress. Monitoring during the prenatal period is sufficient until the time for definitive treatment after childbirth.^11^^,^^12^^,^^18^^,^^20^^,^^54^

Studies have shown that pregnant women with invasive cervical carcinoma have a better prognosis than non-pregnant women do. On the other hand, such findings have been correlated with the larger number of diagnoses during the early stages of pregnancy. When the cases were matched according to staging, the characteristics of the course of the disease, survival rates and complications from treatment were the same.^5^^,^^7^^,^^10^^,^^15^

The maternal prognosis does not seem to be affected in cases of a diagnosis made after 16 weeks of pregnancy with postponement of treatment in order to wait for fetal pulmonary maturity. The rate of recurrence in these cases is similar to that observed among non-pregnant women.^4^^,^^9^^,^^11^^,^^35^^,^^36^

The fetal prognosis is influenced by the type of treatment and by the choice of time when the treatment should be performed, since neonatal morbidity and mortality are related to prematurity.^5^^,^^10^^,^^24^^,^^44^^,^^55^ Even in cases of diagnosis during the third trimester, with the option of immediate treatment, the neonates had greater morbidity and mortality than among other premature neonates of the same gestational age, since infants born to mothers with cervical carcinoma had lower birth weight and higher risk of death on delivery. In addition, the fetal prognosis also depends on intrauterine exposure to cytotoxic drugs, thus demonstrating the importance of adequate prenatal and perinatal care provided by a group with experience in such cases, at a high-risk unit.^5^^,^^9^^,^^10^^,^^34^

## CONCLUSION

Pregnancy is an excellent opportunity for detection of preneoplastic lesions and tumors in the early stages. All patients with abnormal cytological test results should be subjected to colposcopy, which will indicate whether a biopsy is needed and what site is best for performing it. Colposcopy can also rule out or confirm the presence of microinvasion or invasion, thereby defining the type of treatment and the delivery timing and route. Conization in pregnant women is reserved for patients with suspected invasion and is associated with high rates of complications.

Precursor lesions for cervical carcinoma and *in situ* carcinoma should be monitored during pregnancy and reevaluated after delivery, which may be done vaginally.

For microinvasive carcinoma, there are no reports in the literature regarding the best approach or the time for treatment and type of delivery. When the lesion is diagnosed up to the 14^th^ week of pregnancy, conization seems to be the best procedure. After this time, delivery should be awaited. This could be via the vaginal route if the conization margins are disease-free. If conization has not been performed or if the margins are involved, cesarean section should be the choice for delivery route.

In cases of invasive carcinoma detected up to the 12^th^ week of pregnancy, patient treatment is the priority. When a diagnosis is made in the second trimester, it is possible to wait until fetal pulmonary maturity. Many recent studies have indicated the use of chemotherapy in order to stabilize the disease until the time of delivery, which should preferentially be by cesarean section.
